# Rhesus macaques form preferences for brand logos through sex and social status based advertising

**DOI:** 10.1371/journal.pone.0193055

**Published:** 2018-02-20

**Authors:** M. Yavuz Acikalin, Karli K. Watson, Gavan J. Fitzsimons, Michael L. Platt

**Affiliations:** 1 Graduate School of Business, Stanford University, Stanford, CA, United States of America; 2 Institute of Cognitive Science, University of Colorado Boulder, Boulder, CO, United States of America; 3 Fuqua School of Business, Duke University, Durham, NC, United States of America; 4 The Wharton School, University of Pennsylvania, Philadelphia, PA, United States of America; 5 Department of Neuroscience, Perelman School of Medicine, University of Pennsylvania, Philadelphia, PA, United States of America; 6 Department of Psychology, University of Pennsylvania, Philadelphia, PA, United States of America; Centre national de la recherche scientifique, FRANCE

## Abstract

Like humans, monkeys value information about sex and status, inviting the hypothesis that our susceptibility to these factors in advertising arises from shared, ancestral biological mechanisms that prioritize social information. To test this idea, we asked whether rhesus macaques (Macaca mulatta) show choice behavior that is similar to humans in response to sex and social status in advertising. Our results show that monkeys form preferences for brand logos repeatedly paired with images of macaque genitals and high status monkeys. Moreover, monkeys sustain preferences for these brand logos even though choosing them provided no tangible rewards, a finding that cannot be explained by a decision mechanism operating solely on material outcomes. Together, our results endorse the hypothesis that the power of sex and status in advertising emerges from the spontaneous engagement of shared, ancestral neural circuits that prioritize information useful for navigating the social environment. Finally, our results show that simple associative conditioning is sufficient to explain the formation of preferences for brand logos paired with sexual or status-based images.

## Introduction

Despite continued debate about their appropriateness, depictions of sex and social status in advertising continue to help marketers sell their products. Social rewards, including depictions of sex and social status, reliably elicit motivational drives, and their use in advertising is a favored strategy in marketing [1]. Consumers respond more favorably to ads that feature attractive models [2], female nudity in advertising elicits strong physiological responses [3], and sexually explicit content in advertisements increases brand purchase intent [4]. Similarly, the association of products with an individual of high social status is a common advertising strategy, and in some cultures, more than half of all advertisements feature celebrities [5]. Notably, by activating status motives, social status depictions in advertising influence how consumers behave in a variety of contexts, including an increased willingness to pay for larger, more imposing, luxurious, and prestigious products [6–8]. Intuitively, the psychology underlying the formation of advertising-driven brand preferences could have deep evolutionary roots.

Yet, the origins of the effectiveness of sex and status in advertising remain a puzzle. Some argue that our responses to sex and social status are shaped by experience within a particular set of cultural norms, while others argue that such responses reflect the engagement of evolutionarily ancestral mechanisms that prioritize useful social information [9, 10]. Here we build upon prior work to expand our understanding of how consumers respond to depictions of sex and social status in advertisements from an evolutionary perspective. We do this by focusing on whether implicit biases that explain responses to such primitive urges in advertising are shared between humans and our primate cousins [6, 11].

We studied the choice behavior of rhesus macaques (Macaca mulatta) in a laboratory colony. Rhesus macaques are Old World Monkeys native to Central and South Asia. They aggregate into large mixed-sex social groups, in which they form strict dominance hierarchies. Their frequent and highly complex social interactions make them an ideal model species for understanding social cognition and behavior [12, 13]. Rhesus monkeys are highly social, attend to others to gather information, show rudimentary understanding of the intentions of others, care for kin, may give up rewards to alleviate pain in others, and have a genetic basis for some social behaviors [13–20]. Many social behaviors in monkeys are shaped by the same neural mechanisms—often termed the “social brain”—that have been shown to shape social decision making in people [13, 21–23].

Prior studies [15] demonstrated that monkeys will forego fruit juice rewards to view socially-relevant images such as photos of the genitals of males and females and the faces of high status males, suggesting these images have intrinsic value for the animals. An analogous study [24] found that American college students will forego monetary rewards for a glimpse of attractive members of the opposite sex, will wait longer to view attractive individuals than unattractive ones, and will work harder to view more attractive individuals, without being aware they are doing so. These findings suggest that people and monkeys spontaneously, and perhaps subconsciously, value socially-relevant information about others. Endorsing this idea, neurons in reward-related brain areas respond to photos of attractive conspecifics in both people and monkeys [25–27].

Together, these observations invite the hypothesis that receptivity to sex and status in advertising arises from spontaneous activation of neural circuits that prioritize social information in association with neural circuits that process brand information. Associations between a product, a social reward, and the cognitive and physiological state this reward induces in the consumer alone may be sufficient to bias preferences toward the product [28]. Through repeated pairings, brand information, including logos, would eventually become prioritized just as social stimuli are, through the process of conditioning [29]. This simple hypothesis remains to be tested directly in the context of responses to sex and status in advertising. This is an important question, as we do not know whether simple conditioning with socially salient stimuli is sufficient to induce preferences for otherwise neutral logos in the context of sex- and status-based advertising, or whether a more complex, culturally-bound decision process found only in humans is necessary to produce this behavior. In other words, can simple conditioning alone lead to preferences for brands with sexual or social-status based associations? Or, are culturally rooted, complex, and uniquely human mechanisms such as effects of sexual or status-based images on self-concept or selective perception necessary for the formation of such preferences [30, 31]? Because Old World monkeys and humans diverged twenty-five million years ago, the presence of this behavior in both species would suggest evolutionarily ancient origins. Shared brain circuits mediating social perception and valuation in rhesus macaques and humans provide a mechanism by which increased valuation of stimuli associated with social information may be translated into preferences for brands associated with sex and status in advertising [22, 32, 33].

This specific participant population provides us with unique opportunities for a controlled experimental design to test our predictions. Our population of monkeys, due to their lack of experience with advertising, affords the opportunity to create conditioned associations between a brand logo and depictions of sex or social status, the processing of which cannot be distorted by uncontrollable previous, simultaneous, or expected future advertising and brand exposure and experience. Moreover, physical separation of the male and female colonies mitigated any direct influence of the behavior of one sex on the other, which is practically impossible to control for in human participants in an experimental setup. Finally, because monkeys will perform hundreds of trials requiring them to look at advertisements and choose between brand logos for a small drop of fruit juice, we are able to measure behavior at higher levels of engagement than is typically possible in humans inured to advertisements shown repeatedly in a lab environment.

Here we show that in a pseudo-advertising campaign in which brand logos were paired with images of high or low-status male monkey faces, or female monkey genitals, monkeys develop preferences for logos paired with female monkey genitals through conditioning. We found a similar conditioning effect for high-status male faces but not for low-status male faces in both sexes. These findings suggest that sexual and social-status based appeals in advertising are not only processed peripherally, as suggested by prior research [34, 35], but also that associative conditioning driven by prioritization of sex- and status-based social information is sufficient to induce preferences for otherwise neutral brand logos. Further, the negative value of social information in low-status appeals is not sufficient to induce a conditioned withdrawal from neutral brand logos. In other words, preferences for brands advertised with sex or status can emerge without culturally-bound, complex decision mechanisms found only in humans.

## Materials and methods

All procedures in this experiment were conducted in accordance with the PHS Guide to the Care and Use of Laboratory Animals and approved by the Duke University Institutional Animal Care and Use Committee.

### Subjects and housing

Participants were ten adult rhesus macaques (Macaca mulatta; N = 10, 5 females) tested with computers equipped with touch-screens. Monkeys were fed primarily with nutritionally balanced biscuits, with additional daily supplements of fruit, nuts and seeds for enrichment. Access to water was regulated prior to experimental sessions conducted by the lab in order to maintain task motivation for experiments that use fruit juice as reward. If participating in such studies, animals received a minimum 20 mL/kg of water a day with the opportunity for more during the task. When an monkey was not tested for more than five days it was provided at least 50 mL/kg of water a day. During longer periods without participating in a study, animals were permitted free access to water. These amounts were consistent with their daily fluid requirements [36–38]. Still, each animal on regulated access to fluids was also observed daily for health status and hydration. Hydration status was assessed by general appearance (bright, alert, responsive), body weight, skin turgor, and fecal output or consistency by members of the laboratory and veterinary staff.

Male and female colonies were housed separately in two different rooms, each containing approximately twelve to fifteen individuals, at Duke University Vivarium. All monkeys were kept in standard Primate Products NHP Apartment Modules (cage dimensions: 33.25” × 38.5” × 32.75”), had visual and auditory access to all other members in the colony room, and had periodic access to enrichment devices and large play cages. All females were housed in pairs, while all males were housed individually during the timeline of this experiment, due to difficulties finding compatible pair mates. In order to minimize interference from other monkeys during task performance, each participant was confined to a single enclosure during testing.

### Stimuli

**Fig 1:**
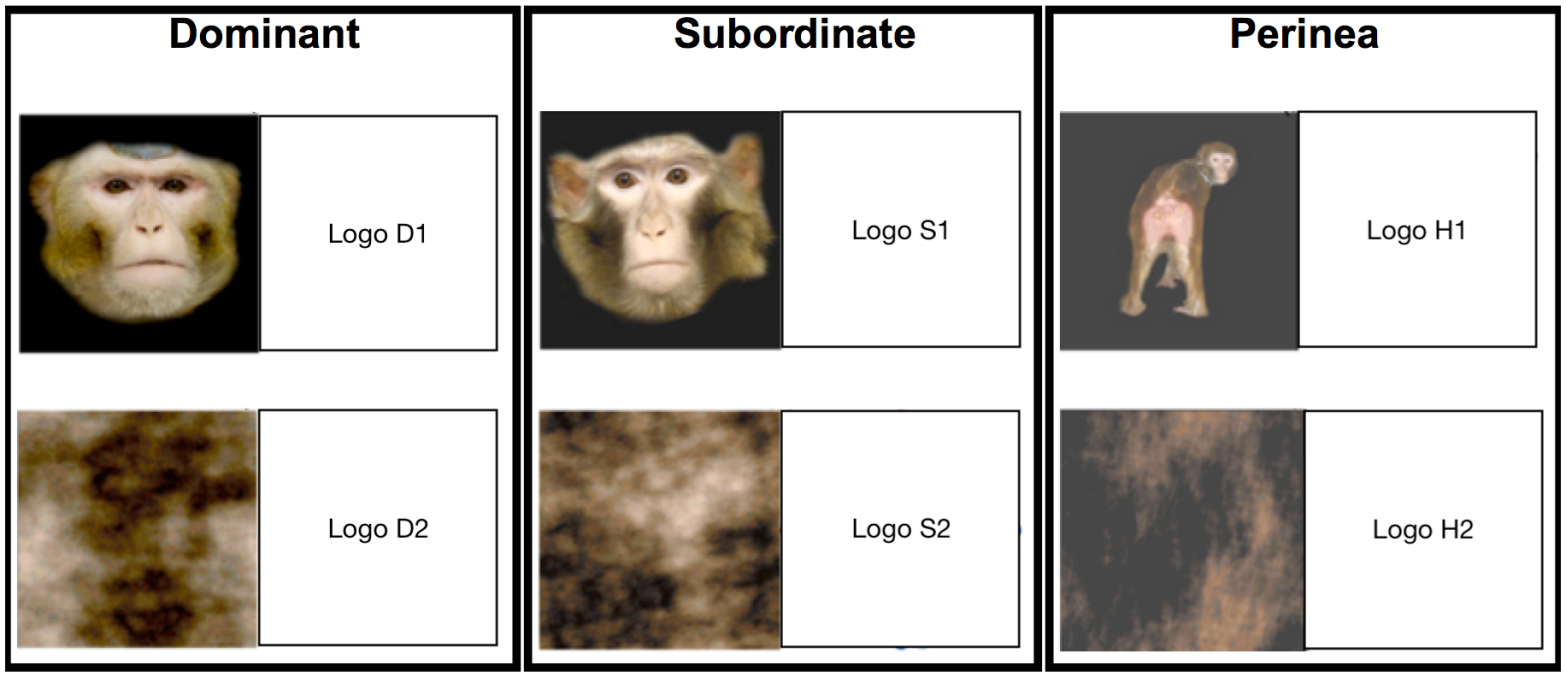
Stimuli examples. Examples of image—logo pairs in order of advertisement category and social image content: Sport: Hindquarters, Car: Dominant, and Pizza: Subordinate. During advertisement trials, images paired with Adidas (Logo H1), Acura (Logo D1), and Pizza Hut (Logo S1) logos included social content (top row; social advertisements), whereas Nike (Logo H2), Citroen (Logo D2), and Domino’s Pizza (Logo S2) logos were paired with scrambled control images (bottom row; control advertisements). The actual study material depicted the brand logos, this version excluding the copyrighted brand logos is intended for illustrative purposes only.

Three sets of stimuli containing images of monkeys from the colony were used as models in an advertising campaign in which social images (female hindquarters, dominant male faces, and subordinate male faces) or their corresponding control images were paired with brand logos to form advertising categories (Fig 1). None of the females were actively menstruating at the time of image collection. Within each trial category, one brand logo was paired with intact social images (e.g. hindquarters, dominant, or subordinate; social advertisements) and the other brand logo was paired with scrambled images (control advertisements). Scrambled control images retained the low-level visual characteristics of the social images, such as luminance and contrast, in absence of coherent object-level information such as whether or not the image was a face or its identity.

### Experimental design

**Fig 2:**
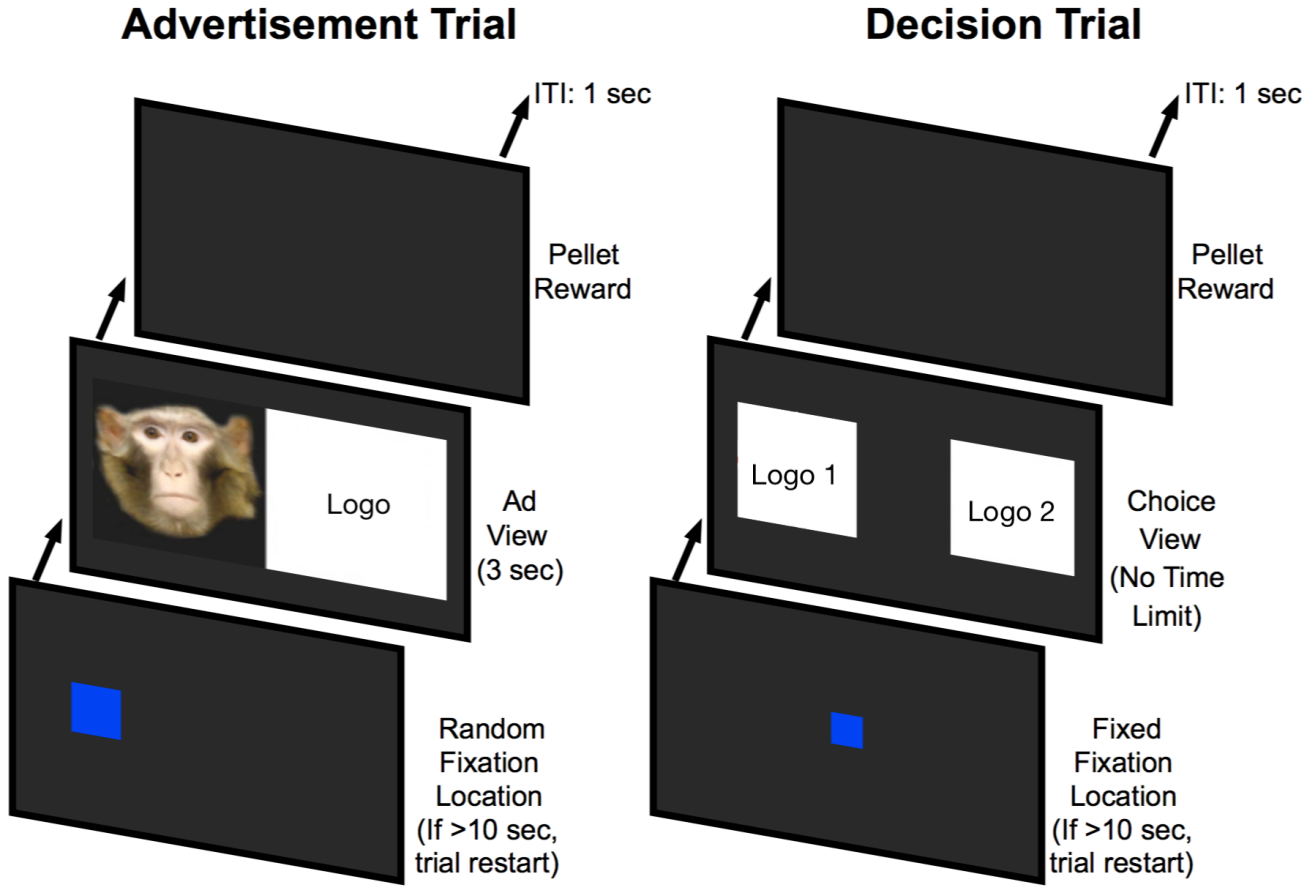
Trial examples. Two example trials. During an advertisement trial, monkeys were required to tap on a randomly placed blue square to see a random advertisement. During a decision trial, monkeys were required to tap on a central fixation square, and then tap on a brand logo image of their choice (both logos were from the same category; Hindquarters, Dominant, or Subordinate). Advertisement and decision trials were randomly interleaved. Only decision trials were used to assess brand logo preferences. The actual study material included the brand logos, this depiction is intended for illustrative purposes only.

Monkeys performed the experiment by interacting with a touch-screen interface within their home enclosures. All monkeys were trained to use a touch-screen prior to the experiment. During each experimental session, monkeys were exposed to a series of advertisement trials that paired a logo with its corresponding social or control image (All logo pairs were pretested to ensure that monkeys did not have preferences among the logo pairs based on their visual characteristics alone, see S1 File). These advertisement trials were randomly interleaved with decision trials, where only the logos that were paired with social images in advertising trials were present (Fig 2).

To initiate advertisement trials, monkeys had to touch a start button that appeared at a random location on the screen. The randomization of the initiation square was to ensure that the monkey was paying attention to the visual information on the screen, so that tapping the screen randomly would not allow moving forward in the experimental procedure and receiving rewards. Advertisements were displayed on the screen for three seconds. At image offset, a small food reward (M&M or Reese’s Pieces) was delivered by an automated reward distribution system to a food hopper centrally located at the bottom of the monitor. The inter-trial interval (ITI) was one second.

For decision trials, the monkey had to touch a start button appearing centrally on the screen. The central location of trial initiation was used to ensure that the subject’s finger would not be spatially biased towards a particular logo at the time of image onset. After the initial screen press, a pair of brand logos (social vs. control logo) corresponding to a particular advertisement category appeared in diametrically opposed locations on the screen. When the monkey selected a logo, he or she received a food reward. Importantly, all rewards were intended to motivate the monkeys to perform the task and to maintain attention on the presented stimuli and were identical across all types of completed trials. There was no correct choice, thus the monkey received an identical food reward regardless of their choice.

The orders of presentation for advertisement and decision trials were drawn randomly with replacement. For decision trials, the ordering of social vs. control logos on the screen (right-left) was also randomized. Each session consisted of 70 ad trials and 30 decision trials. Each monkey completed three sessions, each on different days and each consisting of approximately 100 total trials. This corresponded to a total of 210 advertisement and 90 decision trials (30 per category, non-blocked random assignment) per monkey.

## Results

To test our predictions that (1) monkeys will form socially-conditioned brand preferences, and (2) will respond to repeated advertisement exposure we performed regressions of the binomial choice outcomes (0: chose control brand, 1: chose social brand) on trial category, participant sex, and their interaction using a generalized linear mixed effects model, with intercept-only random effects terms for each monkey. We also controlled for ad repetition by including advertisement repetition counts for social logos, and the difference between the number of social and control logos seen in each category before each trial. We further performed a set of Chi-squared tests comparing choice proportions to indifference to check for robustness and to break down our results further.

**Fig 3:**
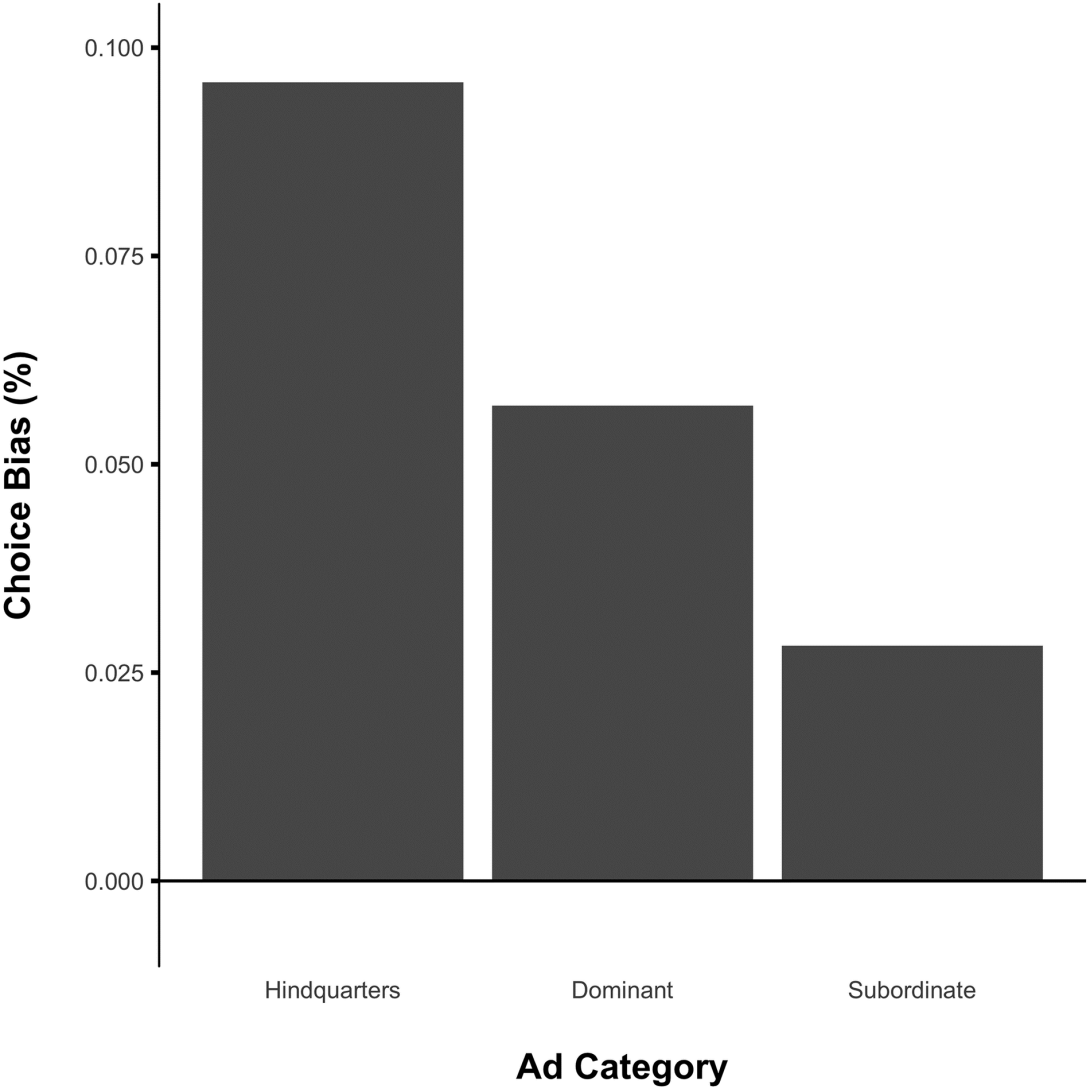
Choice proportions across ad categories. A logistic regression analysis of choices of social and control brand logos revealed that social brand logos were less likely to be chosen in the subordinate category compared to hindquarters (*β*_*HQvsSub*_ = 0.26, z = 2.22, p = 0.026) and dominant (*β*_*DomvsSub*_ = 0.22, z = 1.89, p = 0.059) categories, as well as both (*β*_*SubvsRest*_ = -0.16, z = -2.38, p = 0.017). There was no significant difference in the likelihood of a social brand logo being chosen over a control logo across the hindquarters and dominant categories (*β*_*PerivsDom*_ = 0.042, z = 0.355, p = 0.72).

A generalized linear model analysis revealed that the social brand logo was significantly less likely to be chosen in the subordinate category compared to the other two categories, either controlling (*β*_*SubvsRest*_ = -0.16, z = -2.38, p = 0.017) or not controlling for number of social advertisements seen and the difference in number of advertisements seen for the social and control logos in all categories (*β*_*SubvsRest*_ = -0.16, z = -2.34, p = 0.019). Social brand logos were significantly more likely to be chosen over the control logos in the hindquarters category compared to the subordinate category, controlling (*β*_*HQvsSub*_ = 0.26, z = 2.22, p = 0.026) or not controlling (*β*_*HQvsSub*_ = 0.26, z = 2.24, p = 0.025) for ad repetition. Moreover, social brand logos were marginally more likely to be chosen in the dominant category compared to the subordinate category, again controlling (*β*_*DomvsSub*_ = 0.22, z = 1.89, p = 0.059), or not controlling (*β*_*DomvsSub*_ = 0.21, z = 1.79, p = 0.073) for ad repetition. The likelihood of the social brand logo being chosen over the control logo was not significantly different across the hindquarters and dominant conditions, controlling (*β*_*PerivsDom*_ = 0.042, z = 0.355, p = 0.72) or not controlling (*β*_*PerivsDom*_ = 0.054, z = 0.459, p = 0.65) for advertisement repetition (See S1 Table for regression table). These results provide evidence that monkeys form preferences for brand logos in response to more valuable and socially-informative images associated with brand logos in advertisements (hindquarters and dominant faces but not subordinate faces, see Fig 3).

**Fig 4:**
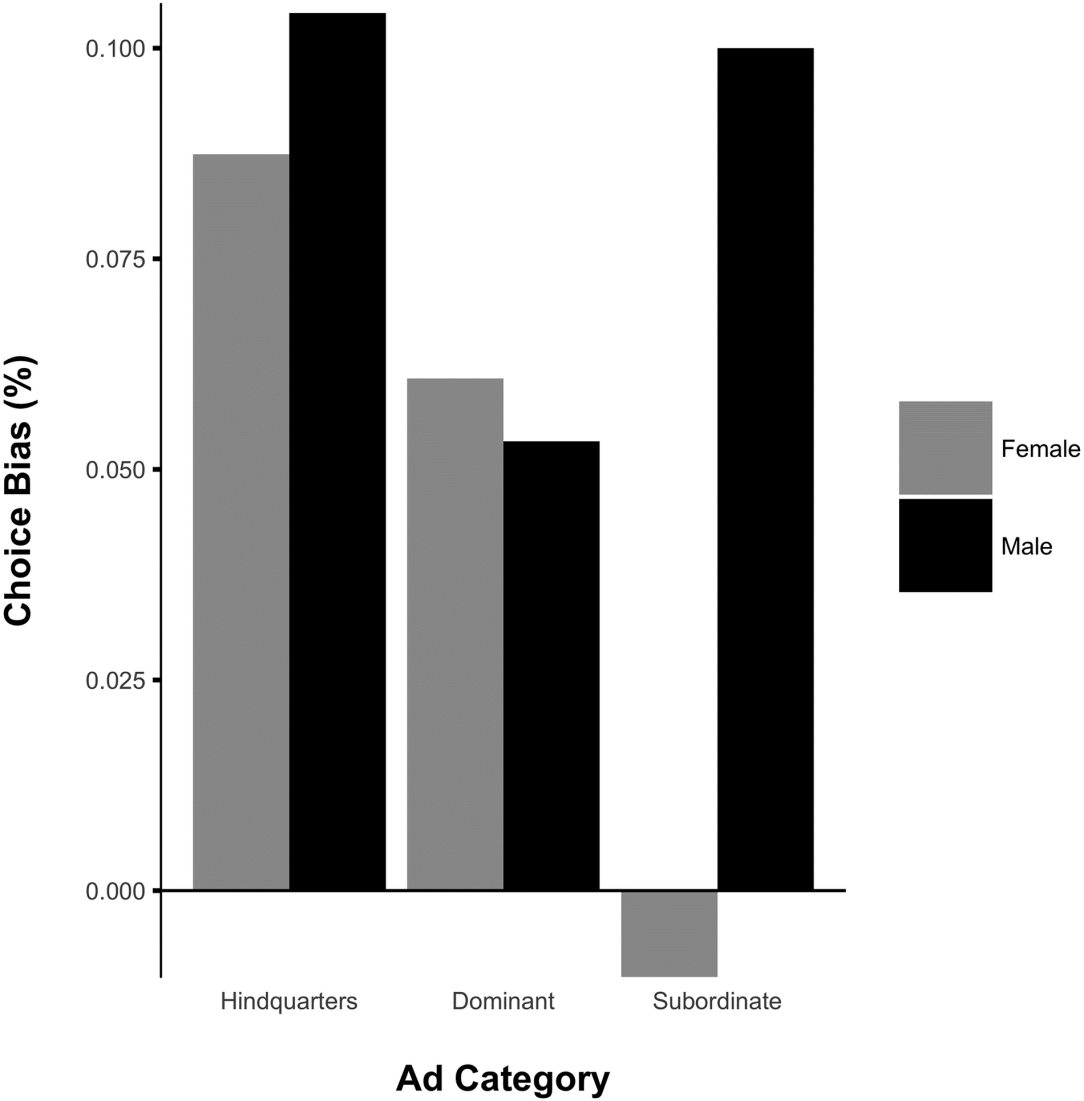
Choice proportions across ad categories broken down by sex. Final choice proportions plotted as percentage bias from indifference (50%). Positive values indicate a preference for the social logo. For the hindquarters category, monkeys were more likely to choose the social logo over the control logo overall (All: *χ*^2^(1, 286) = 10.54, p = 0.0012). For the dominant category, monkeys were also more likely to choose the social logo over the control logo (All: *χ*^2^(1, 297) = 3.89, p = 0.049). For the subordinate category, only males were significantly more likely to choose the social brand over the control brand, whereas females were indifferent to the subordinate social logo (Males: *χ*^2^(1, 150) = 6.00, p = 0.014; Females: *χ*^2^(1, 148) = 1.12, p = 0.29).

To further break down our results and check for robustness, we conducted Chi-squared tests of choice proportions. Monkeys were more likely to choose the social logo over the control logo in the hindquarters category (*χ*^2^(1, 287) = 10.54, p = 0.0012). Furthermore, separated by sex, both male and female monkeys were significantly more likely to choose the social brand over the control brand overall in the hindquarters category (Males: *χ*^2^(1, 144) = 6.25, p = 0.012; Females: *χ*^2^(1, 143) = 4.37, p = 0.037). For the dominant category, monkeys were also more likely to choose the social logo over the control logo (*χ*^2^(1, 298) = 3.89, p = 0.049). When separated by sex, neither males nor females showed final choice proportions that were significantly different from indifference in the dominant category (Males: *χ*^2^(1, 150) = 1.71, p = 0.19; Females: *χ*^2^(1, 148) = 2.19, p = 0.14). This is perhaps due to the reduction in statistical power when samples were subsetted by sex, but overall, both male and female monkeys were more favorable towards the social brand (see Fig 3). Finally, for the subordinate category, monkeys were indifferent between social brand and control brand logo (*χ*^2^(1, 301) = 4.07, p = 0.33). Analyzed by sex, we found a significant preference in males for the social brand over the control brand in the subordinate category, and no significant preference in females (Males: *χ*^2^(1, 150) = 6.00, p = 0.014; Females: *χ*^2^(1, 151) = 1.12, p = 0.29; See Fig 3). These results suggest that overall, monkeys formed preferences for logos paired with images of hindquarters and dominant faces but not subordinate faces following an advertising campaign. Nevertheless, contrary to our predictions, male monkeys formed a preference for the logo paired with subordinate faces as well, rejecting our prediction that both sexes should respond negatively and form preferences against the social logo in the subordinate category. This revealed preference for the subordinate social logo in males could reflect a preference for colony-relevant information instead of a preference for an association with subordination. Since all male monkeys were familiar with each other within their colony, they could have recognized each other as the subordinate models, and there is adaptive value in paying attention to subordinate males who may attempt to advance through the hierarchy through aggression or subterfuge [39]. On the other hand, females formed preferences that were directionally in line with our hypothesis about the subordinate category (see Fig 4).

Finally, we conducted post-hoc analyses related to response time data. Because response times were right-skewed, we log-transformed response times. We a found that response times got significantly shorter as a function of number of advertisements seen in hindquarters (*β*_*nHQAds*_ = -0.00291, t = -2.018, p = 0.044) and dominant (*β*_*nDomAds*_ = -0.00335, t = -2.434, p = 0.015) categories, but not in the subordinate category (*β*_*nSubAds*_ = -0.00159, t = -1.13, p = 0.26), controlling for the difference in number of social and control advertisements seen. These results were consistent when not controlling for the difference in number of social and control advertisements seen as well (*β*_*nHQAds*_ = -0.00285, t = -2.032, p = 0.043; *β*_*nDomAds*_ = -0.00336, t = -2.446, p = 0.015; *β*_*nSubAds*_ = -0.00229, t = -1.79, p = 0.073). These results likely indicate practice effects, as monkeys were becoming more familiar with using the touch-screen as an experimental interface throughout this experiment.

Further, in addition to looking at the effect of reponse times alone, we also included response time metrics in the regressions reported above. Longer response times were associated with increased likelihood of the social brand logo being chosen (*β*_*RT*_ = 0.564, z = 3.151, p = 0.00163). We found that this result was consistent with other relevant varibles included in the regression model (see S2 Table for regression results), and the coefficients on other independent variables remained consistent with the values observed in S1 Table. These results show that our findings are robust to controlling for response times. Longer response times increasing the likelihood of social brand logo choices may indicate, consistent with our hypotheses, that increased amount of processing of the images in decision trials increase choices of the social logos.

## Discussion

Our goal in this study was to explore whether rhesus macaques show choice behavior that is similar to humans in response to sex and social status in a pseudo-advertising campaign. Implicit to this exploration was the question of whether an evolutionarily conserved tendency to value social information is sufficient to induce conditioned responding towards neutral stimuli (brand logos), and to bias preferences between multiple arbitrary stimuli. Our findings show that monkeys develop preferences for common brand logos after a social pseudo-advertising campaign. The advertising-induced preferences depend on both participant sex and advertisement content. Overall, the data provides compelling evidence that some types of social information can be used to induce preferences for brand logos in nonhuman primates, just as in humans. These results highlight the possibility that simple conditioning may drive the effects of sex and status in advertising in humans.

In summary, monkeys formed preferences for brand logos that were paired with images of female hindquarters and the faces of dominant males, but not for brand logos that were paired with the faces of subordinate males. These findings can be informative for understanding responses to depictions of sex and social status in humans [9, 10, 40]. Although our results endorse an evolutionary perspective on why we respond to sex and social status, especially in advertisements, this does not mean that socialization and culturally-specific sex roles have no influence on human choice behavior. In fact, in our experiment, we shaped brand logo preferences via conditioning by using socially rewarding images. We know that social and non-social rewards elicit similar activity in shared neural circuits aross macaques and humans [25–27], and we know that these reward representations can get transferred to the cues that predict them [29]. For this reason, we only argue that in humans there is likely a strong biological drive behind behavioral responses elicited by appeals to sex and status, which may be amplified or muted by social experience. This interpretation of our findings is congruent with previous research suggesting that human preferences for abstract fractal shapes can be manipulated through conditioning with facial images of attractive conspecifics [41].

Our findings support the hypothesis that neural mechanisms that prioritize information about sex and status are shared between macaques and humans. Though adaptive benefits of these mechanisms were realized through the enhancement of survival and reproduction in social groups, and resulting behavioral proclivities continue to shape consumer behavior today, sometimes to the detriment of the pursuit of happiness or profit maximization [6]. Although more elaborate social structures and more complex cognition in humans allow sophisticated rationalizations for brand preferences, those preferences may, at their core, simply be a result of decision-making mechanisms that evolved to solve pervasive environmental and social challenges [42, 43]. Hence, exposure to social stimuli, particularly sexual stimuli, in persuasive content may condition approach tendencies towards brand-relevant stimuli, thus guiding consumer choices.

Importantly, our monkey participants had no external motivation to choose the social logos, since doing so neither resulted in larger food reward, nor a repeated display of the social image previously associated with a logo. Despite the lack of such external incentives, preferences for the social logos were sustained for dozens of trials across multiple testing sessions. For this reason, a decision mechanism that only takes into account material outcomes, such as food, cannot account for our data. Only a decision process that incorporates prioritization of sex and social-status related stimuli could explain these results.

Our findings are not without their limitations. Methodologically, our experimental design did not include a post-conditioning extinction paradigm, thus we are not able to assess consistency of our results with what would be expected from a subsequent extinction paradigm. Further, we can not account for our results from the lens of estrous cycle information. Based on previous hormonal sampling, estrous cycling in these females was irregular and equivocal (unpublished data), so assessment of estrous cycle during the time of image capture was not possible. However, previous results suggests that interest in conspecific images is not influenced by the amount of redness in the image, which is a signal of estrous in rhesus macaques [15, 27]. Conceptually, our focus here was on advertising-driven biases for arbitrary brand logos, without diving deeper into claiming “brand preferences”. Brands and brand preferences in human contexts are complicated and have features, such as extensibility to other product categories, that we could not assess using monkeys as our participants. Thus, it is safer to interpret the data as advertising-induced bias towards arbitrary stimuli (which all brand logos are for humans as well) and not as “brand preferences” in the more complex sense. Another limitation is the nature of our pseudo-advertising campaign, which involved short-term exposure to a large number of advertisements and choices, and did not capture real-life extensions of advertisement campaigns that involve a history with the brand, monetary payments, and social influences. Finally, as with every cross-species approach to understanding a human phenomenon, there are limitations with respect to how much we can generalize these results to human behavior. Nevertheless, we note that the behavioral nuances and neural mechanisms underlying simple conditioning, both of which have undergone decades of scientific scrutiny in both rhesus macaques and humans, are known to be virtually identical in these two species. Our fundamental finding that social images are sufficient to induce approach behavior towards brand logos in monkeys therefore has important implications for understanding the extent to which human responses to sex in advertising could be driven by similar associations.

## Supporting information

S1 File Baseline results. (PDF)

S1 Table Main regression results. Summary of generalized linear mixed effects regression analysis for predicting social brand logo choice. In addition to the independent variables displayed, intercept-only random effects terms for each monkey were included in these models to account for the repeated-measures nature of the task.
(PDF)

S2 Table Regression results with response time measures. Summary of generalized linear mixed effects regression analysis for predicting social brand logo choice, including response times as a covariate in all models. In addition to the independent variables displayed, intercept-only random effects terms for each monkey were included in these models to account for the repeated-measures nature of the task.
(PDF)

S3 Table Regressions predicting response times. Summary of linear mixed effects regression analysis for predicting response times as a function of advertisement exposure-related variables. In addition to the independent variables displayed, intercept-only random effects terms for each monkey were included in these models to account for the repeated-measures nature of the task.
(PDF)
