# Correction: Rhesus macaques form preferences for brand logos through sex and social status based advertising

**DOI:** 10.1371/journal.pone.0194055

**Published:** 2018-03-05

**Authors:** The PLOS ONE Staff

## Notice of Republication

Incorrect versions of Fig 1 and Fig 2 were published in error. This article was republished on February 26, 2018 to correct for these errors. The publisher apologizes for the errors. Please download this article again to view the correct version.
