# Correction: Rhesus macaques form preferences for brand logos through sex and social status based advertising

**DOI:** 10.1371/journal.pone.0237595

**Published:** 2020-08-06

**Authors:** M. Yavuz Acikalin, Karli K. Watson, Gavan J. Fitzsimons, Michael L. Platt

After publication of this article [1], questions were raised about the use of fluid control in the study protocol. The “Subjects and housing” subsection of the Material and methods [1] includes a description of fluid control and assessment of animals’ hydration status: “Access to water was regulated prior to experimental sessions conducted by the lab in order to maintain task motivation for experiments that use fruit juice as reward. … Hydration status was assessed by general appearance (bright, alert, responsive), body weight, skin turgor, and fecal output or consistency by members of the laboratory and veterinary staff.” However, no fluid or food control (restriction) was used to motivate the animals to perform the task in the *PLOS ONE* study [1]. The fluid control statements were included in the paper as background information on protocols approved for other studies in the lab, some of which involved animals that were also used in the *PLOS ONE* study. Only one monkey used in the *PLOS ONE* study [1] also participated in a concurrent study involving fluid control. In that study, the 20 mL/kg/day level stated in the Materials and methods section was a minimum allowable value, not the level actually used, as recommended in [2]. When fluid control was used with this animal, a gradual taper procedure was employed to find the level of daily fluids that would incentivize the animal to perform tasks within the relevant study protocol for which food treats were used as rewards, as described in [3]. Working with a veterinarian to monitor the monkey’s health and welfare, the individual monkey’s daily fluid access was gradually titrated each week until the necessary performance on the task was reached, within the 20mL/kg/day guidelines. While on fluid control, any signs of dehydration would immediately trigger free access to water; however, no signs of dehydration were ever observed. The authors’ prior work showed that this type of fluid control protocol motivates performance while leaving physiological parameters such as body weight and weight gain unaffected [4].

Participation in the task reported in [1] was completely voluntary and was entirely unrelated to the monkeys’ daily food ration, which was selected by veterinarians only on the basis of healthy weight maintenance and nutritional needs. At the beginning of a session, a touchscreen and food reward dispenser were mounted to the front of the monkey’s home cage. Monkeys always received food rewards for completing trials, but if the monkey refused to participate there was no consequence. The data for this study were collected over the course of approximately 6 weeks in order to collect data from all the animals in the study using only a single touch-screen testing system. Each monkey provided 1 session of baseline data and 3 sessions across 3 days of test data.

Concerns were also raised about individual housing of males used in this study [1]. The study began shortly after the last of the long-term stable male pairs in the colony became unsuitable. At the time, the colony had a total of 8 unpaired males. After several months of unsuccessful efforts to pair these animals, it was decided in collaboration with the veterinarian that continued attempts at pairing were creating undue stress [5]. These animals were then moved to protected contact housing, in which they interacted with one another through visual and auditory modalities within the colony room, separated by protective transparent dividers [5]. All animals maintained in protected contact housing were provided with additional environmental enrichment.

This work was overseen by the Duke University IACUC, inspected by the USDA, and voluntarily inspected by AAALAC. All procedures were subjected to ethical review by the Duke University IACUC, and also by a panel of peer reviewers at the National Institute of Mental Health as part of the authors’ application for grant funding (R01-MH086712) to study social reward and motivation, which supported housing, husbandry, and veterinary care for the monkeys used in [1].
