# Supplementary material for: Rhesus macaques form preferences for brand logos through sex and social status based advertising
Source: PLoS One. 2018 Feb 20;13(2):e0193055. doi: 10.1371/journal.pone.0193055 (PMC5819778; doi:10.1371/journal.pone.0193055)
Supplement: S1 File — (PDF) [file pone.0193055.s001.pdf]

Baseline Results

Choices on the 100 baseline decision trials revealed no strong preexisting bias towards any logo prior to the experiment. Specifically, of the thirty Chi-squared tests we ran (3 per monkey for each trial category) to compare choice frequencies between social and control logos to indifference, only one of the male monkeys showed a preference amongst one logo pair (hindquarters category; $\chi^2(1, 43) = 11$, FDR-corrected $p = 0.03$). The results indicated indifference amongst the remaining logo pairs and monkeys (FDR-corrected $p > 0.30$ for all other—29/30—tests). Given the lack of evidence for pre-existing preferences for logo pairs based on their visual characteristics, we moved on to conduct the main experiment.
