# Supplementary material for: Rhesus macaques form preferences for brand logos through sex and social status based advertising
Source: PLoS One. 2018 Feb 20;13(2):e0193055. doi: 10.1371/journal.pone.0193055 (PMC5819778; doi:10.1371/journal.pone.0193055)
Supplement: S1 Table — Summary of generalized linear mixed effects regression analysis for predicting social brand logo choice. In addition to the independent variables displayed, intercept-only random effects terms for each monkey were included in these models to account for the repeated-measures nature of the task. (PDF) [file pone.0193055.s002.pdf]

	Dependent Variable: Picked Social Logo (0/1)					
	Model 1	Model 2	Model 3	Model 4	Model 5	Model 6
sex|Male	0.015	0.015	0.015	0.206	0.206	0.206
	(0.165)	(0.165)	(0.165)	(0.144)	(0.144)	(0.144)
trialcat|PeriVsRest	0.102			0.106		
	(0.069)			(0.069)		
trialcat|DomVsSub	0.221†			0.209†		
	(0.117)			(0.117)		
trialcat|DomVsRest		0.060			0.052	
		(0.068)			(0.068)	
trialcat|PeriVsSub		0.264*			0.264*	
		(0.119)			(0.118)	
trialcat|SubVsRest			-0.162*			-0.158*
			(0.068)			(0.067)
trialcat|PeriVsDom			0.042			0.055
			(0.120)			(0.119)
nDomAds	-0.033†	-0.033†	-0.033†			
	(0.018)	(0.018)	(0.018)			
DomAdDiff	0.033*	0.033*	0.033*			
	(0.016)	(0.016)	(0.016)			
nSubAds	-0.016	-0.016	-0.016			
	(0.018)	(0.018)	(0.018)			
SubAdDiff	-0.005	-0.005	-0.005			
	(0.015)	(0.015)	(0.015)			
nHQAds	0.055*	0.055*	0.055*			
	(0.024)	(0.024)	(0.024)			
HQAdDiff	0.007	0.007	0.007			
	(0.017)	(0.017)	(0.017)			
sex|Male:trialcat|PeriVsRest	-0.071			-0.069		
	(0.098)			(0.097)		
sex|Male:trialcat|DomVsSub	-0.336*			-0.305†		
	(0.167)			(0.165)		
sex|Male:trialcat|DomVsRest		-0.132			-0.118	
		(0.097)			(0.096)	
sex|Male:trialcat|PeriVsSub		-0.274			-0.255	
		(0.169)			(0.168)	
sex|Male:trialcat|SubVsRest			0.203*			0.187†
			(0.097)			(0.096)
sex|Male:trialcat|PeriVsDom			0.062			0.050
			(0.169)			(0.168)
Intercept	0.257†	0.257†	0.257†	0.142	0.142	0.142
	(0.139)	(0.139)	(0.139)	(0.102)	(0.102)	(0.102)
Observations	886	886	886	886	886	886
Log Likelihood	-598.762	-598.762	-598.762	-603.218	-603.218	-603.218
Akaike Inf. Crit.	1,223.524	1,223.524	1,223.524	1,220.436	1,220.436	1,220.436
Bayesian Inf. Crit.	1,285.751	1,285.751	1,285.751	1,253.943	1,253.943	1,253.943
Notes:	$^{*}p < 0.05$, $^{\dagger}p < 0.10$					
