# Supplementary material for: Rhesus macaques form preferences for brand logos through sex and social status based advertising
Source: PLoS One. 2018 Feb 20;13(2):e0193055. doi: 10.1371/journal.pone.0193055 (PMC5819778; doi:10.1371/journal.pone.0193055)
Supplement: S2 Table — Summary of generalized linear mixed effects regression analysis for predicting social brand logo choice, including response times as a covariate in all models. In addition to the independent variables displayed, intercept-only random effects terms for each monkey were included in these models to account for the repeated-measures nature of the task. (PDF) [file pone.0193055.s003.pdf]

	Dependent Variable: Picked Social Logo (0/1)					
	Model 7	Model 8	Model 9	Model 10	Model 11	Model 12
Response Time	0.557**	0.557**	0.557**	0.579**	0.579**	0.579**
	(0.183)	(0.183)	(0.183)	(0.180)	(0.180)	(0.180)
sex|Male	0.034	0.034	0.034	0.229†	0.229†	0.229†
	(0.166)	(0.166)	(0.166)	(0.137)	(0.137)	(0.137)
trialcat|PeriVsRest	0.112			0.115†		
	(0.069)			(0.069)		
trialcat|DomVsSub	0.216†			0.200†		
	(0.118)			(0.117)		
trialcat|DomVsRest		0.052			0.043	
		(0.069)			(0.068)	
trialcat|PeriVsSub		0.276*			0.273*	
		(0.119)			(0.118)	
trialcat|SubVsRest			-0.164*			-0.158*
			(0.068)			(0.068)
trialcat|PeriVsDom			0.059			0.072
			(0.120)			(0.119)
nDomAds	-0.025	-0.025	-0.025			
	(0.019)	(0.019)	(0.019)			
DomAdDiff	0.032*	0.032*	0.032*			
	(0.016)	(0.016)	(0.016)			
nSubAds	-0.018	-0.018	-0.018			
	(0.018)	(0.018)	(0.018)			
SubAdDiff	0.001	0.001	0.001			
	(0.015)	(0.015)	(0.015)			
nHQAds	0.050*	0.050*	0.050*			
	(0.024)	(0.024)	(0.024)			
HQAdDiff	0.010	0.010	0.010			
	(0.017)	(0.017)	(0.017)			
sex|Male:trialcat|PeriVsRest	-0.094			-0.095		
	(0.099)			(0.098)		
sex|Male:trialcat|DomVsSub	-0.310†			-0.271		
	(0.168)			(0.166)		
sex|Male:trialcat|DomVsRest		-0.108			-0.088	
		(0.098)			(0.097)	
sex|Male:trialcat|PeriVsSub		-0.297†			-0.277	
		(0.170)			(0.169)	
sex|Male:trialcat|SubVsRest			0.202*			0.183†
			(0.097)			(0.096)
sex|Male:trialcat|PeriVsDom			0.014			-0.007
			(0.171)			(0.169)
Intercept	0.211	0.211	0.211	0.090	0.090	0.090
	(0.140)	(0.140)	(0.140)	(0.098)	(0.098)	(0.098)
Observations	886	886	886	886	886	886
Log Likelihood	-593.905	-593.905	-593.905	-597.788	-597.788	-597.788
Akaike Inf. Crit.	1,215.810	1,215.810	1,215.810	1,211.576	1,211.576	1,211.576
Bayesian Inf. Crit.	1,282.824	1,282.824	1,282.824	1,249.869	1,249.869	1,249.869
Notes:	†p<0.1; *p<0.05; **p<0.01					
