# Supplementary material for: Rhesus macaques form preferences for brand logos through sex and social status based advertising
Source: PLoS One. 2018 Feb 20;13(2):e0193055. doi: 10.1371/journal.pone.0193055 (PMC5819778; doi:10.1371/journal.pone.0193055)
Supplement: S3 Table — Summary of linear mixed effects regression analysis for predicting response times as a function of advertisement exposure-related variables. In addition to the independent variables displayed, intercept-only random effects terms for each monkey were included in these models to account for the repeated-measures nature of the task. (PDF) [file pone.0193055.s004.pdf]

	Dependent Variable: Response Time							
	RT-1	RT-2	RT-3	RT-4	RT-5	RT-6	RT-7	RT-8
nHQAds	-0.003*			-0.003*			-0.0001	0.003
	(0.001)			(0.001)			(0.005)	(0.006)
nDomAds		-0.003*			-0.003*		-0.005	-0.011*
		(0.001)			(0.001)		(0.004)	(0.005)
nSubAds			-0.002†			-0.002	0.002	0.005
			(0.001)			(0.001)	(0.004)	(0.005)
HQAdDiff				-0.001				-0.003
				(0.003)				(0.004)
DomAdDiff					-0.001			-0.00005
					(0.003)			(0.004)
SubAdDiff						-0.004		-0.008*
						(0.004)		(0.004)
Intercept	0.077*	0.077*	0.077*	0.075*	0.077*	0.083**	0.077*	0.076*
	(0.034)	(0.034)	(0.034)	(0.035)	(0.034)	(0.031)	(0.035)	(0.030)
Observations	886	886	886	886	886	886	886	886
Log Likelihood	-451.174	-450.267	-451.715	-456.127	-455.002	-455.872	-459.216	-470.540
Akaike Inf. Crit.	910.348	908.533	911.430	922.253	920.004	921.745	930.433	959.080
Bayesian Inf. Crit.	929.495	927.680	930.577	946.187	943.938	945.678	959.153	1,002.160

Note: $^{\dagger}p<0.1$; $^{*}p<0.05$; $^{**}p<0.01$
